# Green, Ultrasound-Assisted Extraction for Carvacrol-Rich *Origanum dubium* Extracts: A Multi-Response Optimization Toward High-Value Phenolic Recovery

**DOI:** 10.3390/molecules30234620

**Published:** 2025-12-01

**Authors:** Magda Psichoudaki, Yiannis Sarigiannis, Evroula Hapeshi

**Affiliations:** 1 Department of Health Sciences, Pharmacy, School of Life and Health Sciences, University of Nicosia, Nicosia 2417, Cyprus; sarigiannis.i@unic.ac.cy (Y.S.); hapeshis.e@unic.ac.cy (E.H.); 2 University of Nicosia Research Foundation (UNRF), Makedonitissas Avenue 46, Nicosia 1700, Cyprus

**Keywords:** *Origanum dubium*, carvacrol, green chemistry extraction, Response Surface Methodology (RSM), Box–Behnken design (BBD), pulse-mode ultrasonic-assisted extraction (UPAE), medicinal aromatic plants (MAPs), HPLC

## Abstract

*Origanum dubium,* mainly grown in the Mediterranean region, is one of the less extensively studied species among the oregano class. Oregano species are recognized for their significant pharmaceutical properties, primarily attributed to carvacrol and other phenolic compounds. The goal of this study was to establish a sustainable method for the extraction of carvacrol, total phenolic, and total flavonoid compounds (TPC and TFC, respectively). Pulse-mode ultrasonic-assisted extraction (UPAE) was employed, using ethanol–water mixtures as green solvents, for the extraction of the bioactive compounds from the plant material. A Box–Behnken design (BBD) coupled with Response Surface Methodology (RSM) was applied to optimize the extraction process with respect to the extraction temperature, extraction time, ethanol-to-water ratio of the solvent and power amplitude of the ultrasonic processor. The responses of carvacrol (determined by HPLC-PDA), TPC, and TFC (determined by spectrometric methods) were evaluated by RSM. The statistical model identified the optimal extraction conditions, which were a combination of increased extraction temperature (70 °C) for 26 min with an intermediate ethanol–water ratio (60%) at the maximum processor’s power amplitude (100%). These conditions led to the optimal response of the three measured parameters. The optimized parameters represent a green and efficient approach to obtain bioactive-enriched extracts from *Origanum dubium*, suitable for potential applications in functional foods, preservatives, or other applications.

## 1. Introduction

The genus *Origanum* L., widely distributed across the Mediterranean region, exhibits a large chemical diversity among different species. Phenolic acids and flavonoids from oregano plants have been found to exhibit antioxidant, anticancer, and anti-inflammatory potential [1,2,3]. Due to these properties, oregano has also been assessed as a potential preservation and sanitizing agent [4,5,6,7]. *Origanum dubium Boiss*., an endemic species of Cyprus, has a significant essential oil content (6–8%), and both its essential oil and its extracts are rich in phenolic and other bioactive molecules, making it a promising source of bioactive compounds for nutraceutical applications [4,8,9]. Although the *O. dubium* species remains poorly characterized in terms of its bioactive compounds and secondary metabolites, the major phenolic and flavonoid components that have been detected in oregano species extracts and essential oils are carvacrol, thymol, p-cymene, rosmarinic acid, chlorogenic acid, quercetin, luteolin, and others [10,11,12,13,14,15].

Oregano species are also known to be rich in phenolic monoterpenes, notably carvacrol and thymol, which contribute to the well-documented beneficial properties of their extracts [1,2,3]. Among them, carvacrol, a monoterpenoid phenol, which has been identified as a predominant and probably most important secondary metabolite of oregano, is mainly responsible for the plant’s well-documented beneficial biological (antibacterial, antiviral, antifungal, and anticancer) properties [16,17,18,19,20,21]. Carvacrol also has a well-characterized safety profile and has already been approved by the FDA and the European Commission as a food additive [10,17,22,23]. These attributes have rendered carvacrol as a prominent target compound in both phytochemical and pharmaceutical research [24]. Especially for the food industry, there is an increased interest in natural compounds, including extracts from medicinal aromatic plants (MAPs) [6].

As the bioactivity of a plant extract is strongly related to its concentration in active ingredients, as well as the extractability of the latter from the plant matrix, it is essential to emphasize the development and optimization of robust extraction protocols that can efficiently and reproducibly maximize carvacrol’s and other bioactive ingredients’ (i.e., phenolics and flavonoids) extraction yields from the oregano plant. The selection of the extraction method is a key step in the maximization of the recovery of bioactive ingredients from MAPs, as it affects both the types of secondary metabolites (phenolics, flavonoids, terpenoids) that can be isolated, their amounts, as well as the efficiency of the obtained extracts in potential applications. Conventional extraction approaches for MAPs, such as Soxhlet and maceration, require prolonged heating and large solvent volumes, which can degrade volatile terpenes, impact the stability of bioactive compounds, and increase the environmental impact of the extraction procedure [25,26,27,28]. For example, several studies have shown that prolonged extraction times (>60 or 90 min) may lead to a decrease in the total phenolic content (TPC) of several aromatic plant extracts [29,30]. Consequently, the development of eco-friendly and efficient extraction approaches for oregano species is, therefore, essential to obtain high-quality extracts rich in carvacrol and other key phytochemicals.

More recently, as alternatives to conventional ones, efficient greener extraction techniques have emerged, that align with the principles of green chemistry and sustainable processing [25]. The principles of green chemistry aim to minimize the environmental footprint of chemical processes, in terms of energy consumption, waste generation, and solvent toxicity. Among green extraction methods, ultrasonic-assisted extraction (UAE) has appeared as a sustainable technique for effectively extracting bioactive phytochemicals from different plant matrices. The application of UAE induces cavitation effects that improve solvent penetration by disrupting plant cells, facilitating the extraction of the plant’s chemical constituents into the extraction solvent [31,32]. The implementation of UAE has been shown to enhance mass transfer, leading to decreased extraction times and volume of extraction solvents used, improved extraction recoveries, while requiring lower power consumption [33,34,35,36]. Additionally, probe-type UAE systems, which enable the direct sonication of the plant material, have been shown to often outperform bath-type UAE, providing higher yields for several bioactive compounds [37,38,39]. Pulse-mode UAE (PUAE) represents a modified operation mode of the probe-type UAE, in which the ultrasonic energy is applied intermittently to the sample in the form of pulses rather than continuously. PUAE can provide similar and often better extraction yields, while requiring less energy input [38,40,41].

The efficacy of the extraction procedure also relies on the selection of an appropriate extraction solvent. For phenolic compound extraction, relatively polar solvents are typically selected. Among them, water and ethanol are recognized as environmentally friendly and green solvents and have therefore been increasingly applied, usually combined, for the extraction of MAPs [42,43]. The selection of the ethanol-to-water ratio is crucial, as MAPs have variable composition in phytochemicals and metabolites. Additionally, most phenolic compounds have low aqueous solubility; however, the presence of water in the solvent mixture has been found to improve extraction yields, due to the swelling effect of water on the plant tissues, which facilitates the release of phytochemicals [44].

Based on the above, the selection of the extraction parameters is of major importance, as they directly impact the obtained yields. For the systematic evaluation of extraction parameters, the application of the Box–Behnken Design (BBD) can provide a statistically robust framework [45,46].

Despite extensive extraction optimization works in the literature, several gaps remain: As most studies investigate the impact of solvent, time, and extraction temperature, the sonication parameters are often under-explored. Additionally, for oregano, especially, carvacrol is rarely quantified in optimized extracts. To the authors’ knowledge, currently, no optimized green extraction method specifically targeting carvacrol from oregano has yet been reported in the literature.

Thus, the present study aimed to develop and optimize a probe-type, ultrasound-assisted green extraction protocol for *Origanum dubium*, using an ethanol–water system, targeting the maximization of carvacrol, total phenolic content (TPC), and total flavonoid content (TFC) of the extracts. The tested parameters were the following: solvent’s ethanol-to-water ratio, extraction time, temperature, and sonicator’s power amplitude. The carvacrol responses were quantified using a High-Performance Liquid Chromatography system coupled with a Photo Diode Array detector (HPLC-PDA), TPC was estimated with the Folin–Ciocalteu method, and TFC using the aluminum chloride assay. Finally, the implementation of Response Surface Methodology (RSM) provided optimal extraction conditions. The developed optimized method offers an energy-efficient and scalable process for producing carvacrol-rich, antioxidant extracts; thus, the findings of this work contribute to the increasing number of green extraction protocols for MAPs, while they can be further applied to enable effective recoveries of *Origanum dubium* and other oregano species bioactive compounds for potential nutraceutical or other types of applications.

## 2. Results and Discussion

### 2.1. Selection of Extraction Conditions

Previous studies using ethanol–water mixtures for the extraction of bioactive compounds of oregano showed better extraction yields with intermediate-to-high ethanol-to-water ratios; thus, in this work, the selected tested levels of ethanol content of the extraction solvent ranged from 40% to 80% [47,48,49]. Other organic solvents, especially methanol, have also been proven to be efficient for the extraction of bioactive molecules from MAPs. However, ethanol–water mixtures were selected in this work, as ethanol has lower toxicity and aligns with the principles of green and sustainable chemistry. Adding to its lower environmental hazards, ethanol is also compatible with food and pharmaceutical applications [50]. When ultrasound-assisted extraction (UAE) is applied, long extraction times are normally applied, often up to one hour, for oregano or similar aromatic plant species, but the application of the ultrasonic processor probe has been shown to be more effective and less time consuming compared to bath-type UAE [51]. Additionally, the application of PUAE has been found to increase the recovery of the extracted polyphenols; thus, the factor of time was tested in the range of 5 to 30 min [38,41,52]. Some studies have also shown that the use of sonication probes in pulse mode can improve extraction yields compared with continuous sonication; thus, pulse mode with a 50% duty cycle was selected for these experiments [41,53]. The impact of the sonicator’s power amplitude on the extraction yields has not yet been tested extensively. Kumar et al. (2021) used a probe-type ultrasonic processor for the extraction of bioactive molecules from *Origanum vulgare*, at a selected power amplitude of 80% [44]. In this work, the sonicator’s power amplitude was tested between 50% and 100%. The extraction temperature is also a critical extraction parameter that may significantly affect phenolic compound extraction yields. Elevated temperatures can improve mass transfer from plant material to the solvent but can also degrade some phenols. Typical extraction temperatures previously used for *Origanum vulgare* ranged from room temperature up to 80 °C [38,47,54]. Here, the temperature was tested between 40 °C and 80 °C.

### 2.2. Optimization of Extraction Conditions

#### 2.2.1. Response Surface Analysis of Carvacrol Content

**Figure 1:**
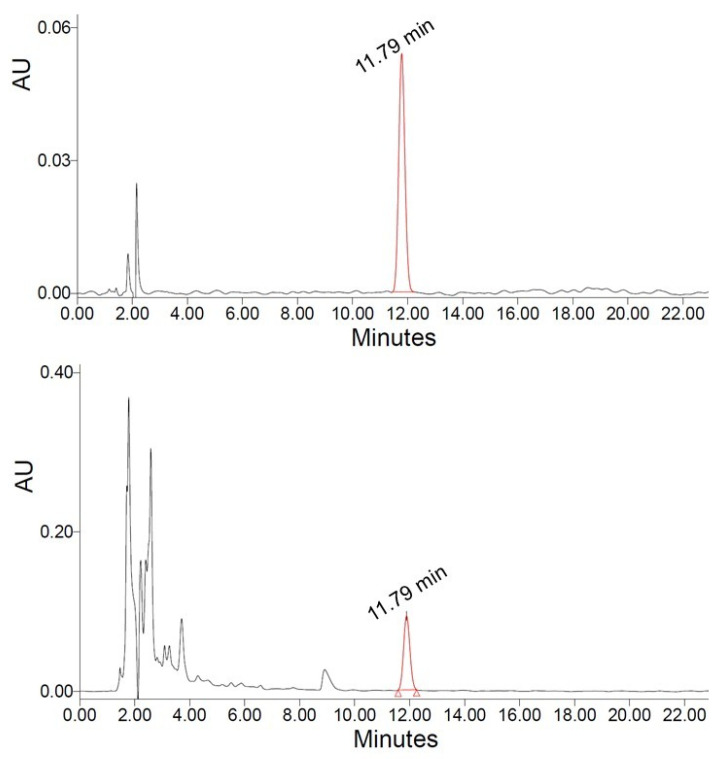
Chromatograms of sample extract (**lower**) and carvacrol standard (**upper**), obtained at 280 nm.

In this study, the carvacrol content of the ethanol–water extracts of *Origanum dubium,* estimated by HPLC-PDA (Figure 1), ranged from 7.68 to 15.89 mg/g of plant dry mass (dm).

**Figure 2:**
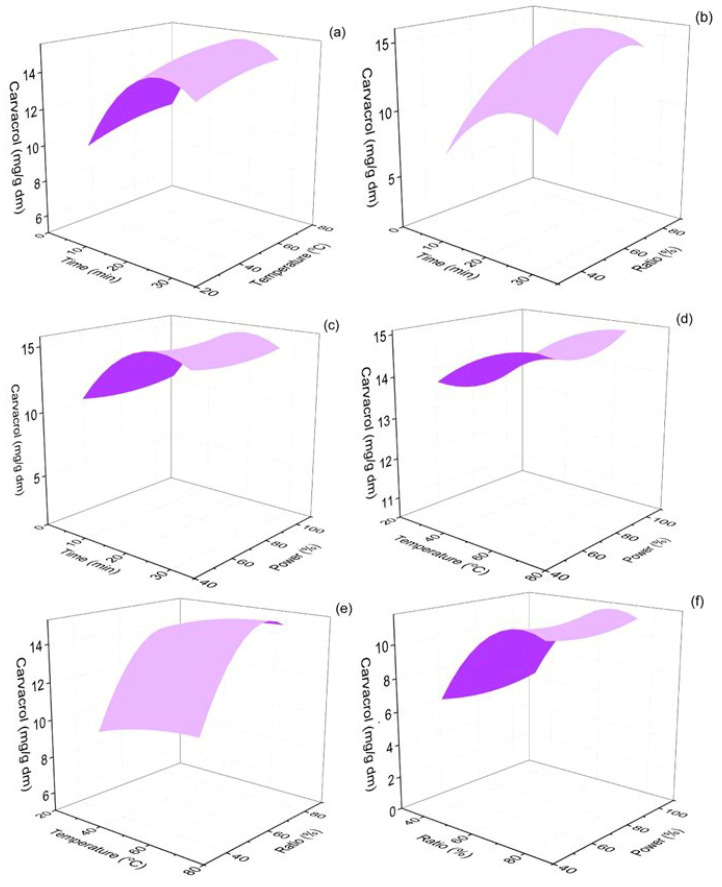
Response surface 3D plots of carvacrol response (in mg/g dm) as a function of extraction time and temperature (**a**), extraction time and ethanol–water ratio (**b**), extraction time and % sonication power (**c**), extraction temperature and % sonication power (**d**), extraction temperature and % sonication power (**e**) and ethanol–water ratio and % sonication power (**f**).

The 3D response surface plots showing the effect of extraction time, extraction temperature, % ethanol–water ratio of the solvent used, and the percentage of the power amplitude of the ultrasonic processor on the levels of the extracted carvacrol are shown in Figure 2. According to Figure 2, carvacrol yield increased with an increase in extraction time, up to ~20 min, while further extension of the extraction led to a slight decrease in the obtained carvacrol content of the extracts. 

The increase in extraction temperature, up to 70 °C, was also found to favor carvacrol extraction. The ethanol content in the extraction solvent also strongly impacted carvacrol yields: high water content led to poor carvacrol extraction from oregano leaves, and its yield maximized at ~70% ethanol content of the solvent mixture. Finally, the amplitude of sonication power showed a negative correlation with carvacrol yield from low to medium power amplitude, but the yield increased with higher amplitudes and reached its maximum at 100% of sonication power intensity. The ANOVA results are presented in Table 1.

Three out of four independent parameters, i.e., extraction time (*p* < 0.001), ethanol ratio (*p* < 0.001), and extraction temperature (*p* < 0.05), were found to significantly influence carvacrol response. These parameters have also been previously reported to be crucial for the efficacy and extraction yields in similar green extraction applications [36]. Pandey et al. (2018) optimized the extraction of phenolics and antioxidant species from *Rheum moorcroftianum,* using ultrasound-assisted sonication, and identified extraction temperature as a significant parameter for the maximization of the targeted parameters (tested temperature range 35–55 °C), while they also found that extraction time was not a significant factor in the tested extraction time range (10–20 min) [55]. Zhang et al. (2009) found similar patterns for the extraction yields of epimedin C from *Epimedium* species as in this study [56]. This work used methanol–water mixtures and also found that the extraction yield increased with an increase in methanol content, up to a specific ratio (~60%), and then decreased with the further organic solvent content increase, similar to the findings of this work (Figure 2b,e,f) [56].

#### 2.2.2. Response Surface Analysis of Total Phenolic Content (TPC)

**Figure 3:**
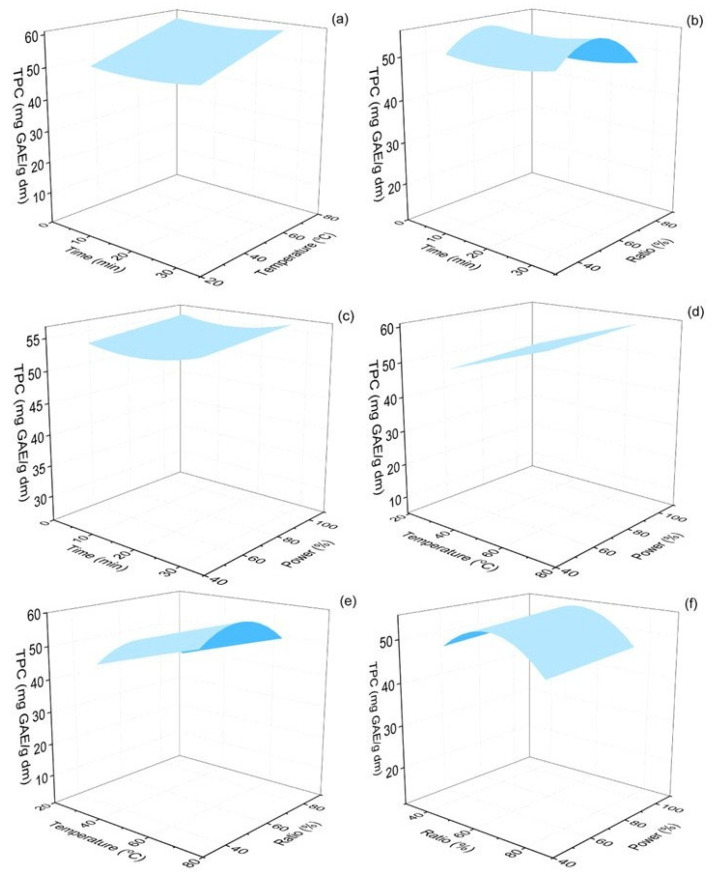
Response surface 3D plots of TPC response (in mg GAE/g dm) as a function of extraction time and temperature (**a**), extraction time and ethanol–water ratio (**b**), extraction time and % sonication power (**c**), extraction temperature and % sonication power (**d**), extraction temperature and % sonication power (**e**) and ethanol–water ratio and % sonication power (**f**).

The total phenolics of the *Origanum dubium* dry leaves extracts ranged from 43.83 to 69.28 mg GAE/g dm. The response surface plots illustrating the effect of the extraction parameters on the levels of the measured TPC are represented in Figure 3. The extraction temperature was found to be significant (*p* < 0.001), with TPC reaching optimal values at elevated temperatures (70 °C). Similar extraction temperatures for TPC were reported by Michalaki et al. (2023), who assessed the optimal extraction conditions for maximized phenolics and antioxidant activity of *Origanum vulgare* leaves [47]. In this study, the tested extraction temperature range was from 40 to 80 °C, while the combined optimal extraction temperature was found to be 80 °C.

The other three evaluated parameters from this work were found to be statistically insignificant for the TPC response. However, the analysis showed that there was an optimal ethanol content of the solvent mixture that maximized the extraction of phenolic compounds (~60%), while lower or higher ratios led to decreased TPC yields (Figure 3). Extended extraction times (up to 30 min) and also high power amplitude of the ultrasonic processor (100%) also favored the increase in the phenolic content of the extracts. These findings are in agreement with the study of Michalaki et al. (2023), who also found that, for the oregano plant, the optimal ethanol ratio of the solvent was 60%, and also that extended extraction times, up to 30–40 min, maximized the TPC content of the extracts [47]. Athanasiadis et al. (2024) also used ultrasonic-assisted extraction to estimate the recovery of TPC from *Rosmarinus officinalis* leaves and found that it maximized at the highest extraction time tested (20 min) [39].

Increased extraction temperatures have been previously shown to assist the extraction of phenolic compounds, due to an increased mass transfer rate and cavitation effect; however, very high temperatures and prolonged extraction times may lead to the degradation of phenolic compounds and flavonoids [44,57]. Oreopoulou et al. (2020) conducted a kinetics study on the extraction of phenolic compounds from *Origanum dubium* plant remains after distillation and also optimized their extraction using ethanol–water mixtures [58]. The findings of this work are in alignment with the results of our study, as they also found that the maximum phenolics yield was achieved with 60% ethanol content of the solvent mixture. Additionally, they evaluated the effect of a temperature increase from 22 to 60 °C and also found that TPC content maximized at the highest temperature of 60 °C. Another work assessed, among others, the impact of the organic solvent ratio of the extraction solvent using ultrasound-assisted extraction and methanol as the organic solvent and observed an increase in TPC in oregano extracts with an increase in the methanol ratio from 40 to 60% [59]. In general, the obtained levels of phenolics acquired in our work are satisfactory and show that the applied extraction method was highly efficient, as the observed TPC levels are comparable or even significantly higher compared with results from similar studies. The estimated TPC is comparable to the value calculated for *Origanum vulgare* ethanolic extracts, obtained with the use of PUAE (63 mg GAE/g dm) from Rout et al. (2021) and higher than the TPC measured in *Origanum dubium* ethanolic extracts (28 mg GAE/g dm) prepared by maceration of plant dry mass [8,38].

#### 2.2.3. Response Surface Analysis of Total Flavonoid Content (TFC)

**Figure 4:**
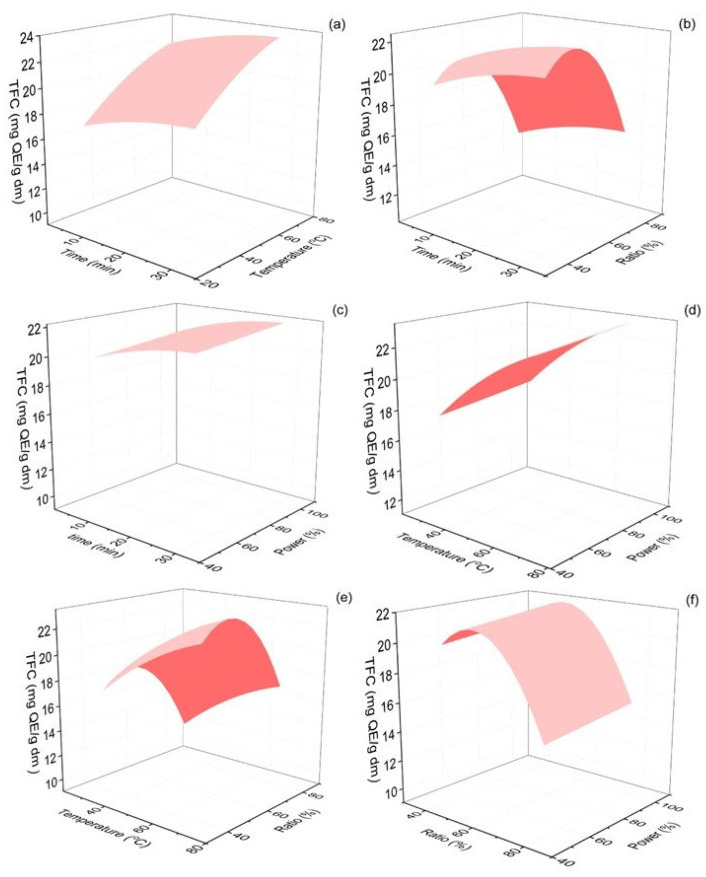
Response surface 3D plots of TFC response (in mg QE/g dm) as a function of extraction time and temperature (**a**), extraction time and ethanol–water ratio (**b**), extraction time and % sonication power (**c**), extraction temperature and % sonication power (**d**), extraction temperature and % sonication power (**e**) and ethanol–water ratio and % sonication power (**f**).

The total flavonoid content of *Origanum dubium* dry plant mass was evaluated according to the method described in Section 3.6. In this set of experiments, TFC ranged from 13.70 to 25.73 mg QE/g dm. The response surface plots presenting the interactions of the examined extraction parameters with the estimated TFC of the extracts are shown in Figure 4. The lack of fit value estimated was 0.21, indicating that the model adequately fitted the experimental data, as the lack of fit was not statistically significant (*p* > 0.05).

As expected, and since flavonoids are a sub-class of the phenolic compounds of oregano plants, the optimal extraction conditions for TPC and TFC were rather similar. For the flavonoids extraction, the extraction temperature and the ethanol content in the extraction solvent were found to be significant parameters (*p* < 0.001). According to Figure 4, an increase in extraction temperature up to 70 °C led to the maximization of TFC in the plant extracts. Regarding the ethanol content of the solvent, an initial increase from 40 to ~53% favored an increase in TFC but further increases in the organic solvent in the mixture led to significantly lower TFC values. Elevated extraction times, up to 30 min, and the use of the ultrasonic processor at the maximum amplitude also positively impacted TFC yields.

These findings are consistent with previous studies, demonstrating that intermediate ethanol–water ratios facilitate the extraction of moderately polar flavonoids. A similar optimal ethanol ratio in the extraction solvent for the maximization of flavonoids was also estimated by Jovanovic et al. (2017), who tested the efficacy of different extraction methods [60]. Solvent ethanol content of 50% was found to favor TFC yields for all three tested extraction methods, i.e., maceration, UAE, and heat-assisted extraction. This solvent shows the potential to solubilize flavonoids, providing an optimal polarity that enhances their diffusion into the extraction solvent.

Rout et al. (2021) also tested the effect of sonication amplitude on the TFC of *Origanum vulgare* leaf sheds and found that increased power amplitude (80%) optimized TFC yield [38]. Kobus et al. (2022), using an ultrasonic processor probe for the extraction of phenolics and flavonoids from *Sorbus intermedia*, and testing extraction times from 5 to 15 min, also observed that an increase in extraction time favored TFC yield [41].

### 2.3. Effect of Process Parameters on Carvacrol, TPF, and TFC Levels of Extracts

**Figure 5:**
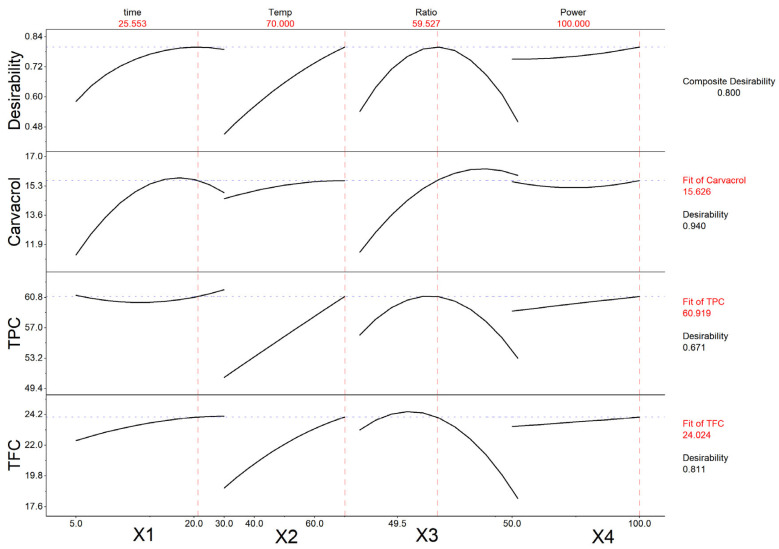
Illustration of the effect of each extraction parameter on the measured characteristics (carvacrol content, TPC and TFC) of the oregano extracts, with the use of an ultrasonic processor probe.

Regression analysis was performed to fit mathematical models to the experimental data, aiming to determine the optimal region within the design space for the evaluated responses. Figure 5 represents the responses of each evaluated parameter as a function of each independent factor applied. The analysis showed that the combined optimal extraction conditions for the *Origanum dubium* plant leaves were the application of 26 min extraction time, at 70 °C, using an ethanol–water solvent of 60% *v*/*v* ethanol, and operating the ultrasonic processor probe at its 100% power amplitude in pulse mode (Table 2).

According to Figure 5, carvacrol yield reaches its maximum before the optimal time of 26 min (at 17.5 min), but the application of a longer extraction time has a minor impact on the carvacrol content of the extracts. Regarding the extraction temperature, all three measured parameters are maximized at high temperatures (70 °C). The ethanol content of the solvent also affects the levels of the measured TPC, TFC, and carvacrol content of the extracts: while TFC and TPC reach maximum levels with solvents containing 53 and 58% *v*/*v* ethanol, respectively, carvacrol extraction is enhanced with the use of solvents with a higher ethanol content (71%). Carvacrol, being a low-polarity monoterpenoid phenol, exhibits limited solubility in a highly aqueous solvent. Consequently, an extraction protocol employing ethanol–water mixtures as an extraction solvent requires higher ethanol–water ratios to effectively recover carvacrol from oregano plant leaves. Still, the presence of water in the solvent at low proportions is beneficial, as it has been shown to improve the extraction of carvacrol [59].

Thus, in the application of PUAE, when the aim of the extraction is to obtain a carvacrol-rich extract, the ethanol content of the solvent should be higher than the combined optimal ethanol ratio, while when the aim is the maximization of all three parameters, the application of the estimated optimal ethanol ratio would be more appropriate. The amplitude of the ultrasonic processor power was not found to significantly impact the levels of the evaluated parameters; still, all three parameters maximized at an amplitude of 100%.

Although the three responses exhibited distinct kinetic and solvent behavior—most notably, carvacrol reaching its maximum yield at a shorter extraction time and higher ethanol-to-water ratio compared with TPC and TFC, they converged in their optimal values at the same temperature and sonication power. The composite desirability value for the optimal conditions was 0.80, indicating a satisfactory compromise among the targeted extraction responses. This suggests that the selected conditions effectively balance the simultaneous maximization of phenolics, flavonoids, and carvacrol content, aligning with the defined optimization criteria.

Table 3 summarizes the optimal extraction conditions for each optimized parameter, as predicted by RSM, and the combined optimal conditions for the maximization of all three evaluated parameters. The table also represents the predicted response values and the corresponding experimental values. The relative error estimated for all measured parameters was lower than 10%, indicating the suitability and efficacy of the model to provide optimal extraction conditions.

The findings highlight the application of BBD and RSM for the optimization of a probe-type, ultrasound-assisted green extraction method, which not only enables the enhancement of the recovery of individual bioactive constituents but also allows for the identification of the process conditions that balance multiple response targets within a single extraction protocol.

## 3. Materials and Methods

### 3.1. Chemicals and Reagents

Water, ethanol, and acetonitrile, used as mobile phases and solvents, were LC-MS grade and purchased from Supelco (Darmstadt, Germany). Carvacrol, gallic acid, and quercetin standards (1 mg/mL) were purchased from LGC (Ausgburg, Germany). Folin–Ciocalteu reagent was purchased from Sigma Aldrich (Steinheim, Germany). AlCl_3_, Na_2_CO_3_, and NaOH were all of analytical grade and were purchased from Merck (Darmstadt, Germany).

### 3.2. Experimental Design

For optimization of the extraction procedure of carvacrol, TPC, and TFC from oregano plant dry mass, RSM was employed. The experimental procedure was conducted based on the application of BBD to evaluate the combined effect of four parameters (extraction time, extraction temperature, % of ethanol content of extraction solvent, % of amplitude of ultrasonic power) using 3 levels per factor (low, center, high). The factor levels used were extraction time: 5, 15, and 30 min; extraction temperature: 30 °C, 50 °C, and 70 °C; ethanol ratio: 40%, 60%, and 80%; and sonication power: 50%, 75%, and 100%.

As four key factors were to be optimized, a 27-run design was used, including three center replicates (X1: extraction time, X2: extraction temperature, X3: ethanol ratio, X4: sonication amplitude). In order to reduce variations in the response values due to external factors, the proposed design produced the 27 runs in a randomized manner. The total number of experiments was calculated based on the following equation:

N = 2k(k − 1) + C_0_

where N is the total number of experiments, k is the number of factors, and C_0_ is the number of central points [61].

Table 2 lists each run with the corresponding independent variables, with coded and actual values, along with the experimental measurements of TPC, TFC, and carvacrol content of the extracts.

### 3.3. Extraction Procedure

The aerial parts of *Origanum dubium* were collected during April of 2025 from cultivated accessions of the Ministry of Agriculture, Rural Development and Environment, Forestry Department (Nicosia, Cyprus, latitude 35°8′7.8101″ N, longitude 33°24′8.0802″ E) and dried at room temperature. The dry plant mass was ground and stored at 4 °C until analysis. For the extraction, 0.50 g of plant dry mass was placed in a Falcon tube with 10 mL of solvent. The extraction was conducted using an ultrasonic probe processor (UP200Ht, Hielscher, Teltow, Germany), 2 cm depth in the sample, operating at a fixed frequency of 26 kHz, and a nominal power output of 200 W in pulsation mode (50% duty cycle). The sonicator was equipped with a titanium sonotrode of 14 mm diameter, and the experiments were carried out in three levels of power amplitude, as described above. During the extraction, a heated water bath was used to maintain the temperature stable (±2 °C) during the extraction procedure. After extraction, plant extracts were centrifuged for 15 min at 10,000 rpm (Centurion Scientific, Rotor BRK 5520, Stoughton, UK), the supernatant was collected, filtered through 0.2 μm PES syringe filters and properly diluted before analysis.

### 3.4. Determination of Carvacrol Content

The carvacrol content of the obtained extracts was determined by the use of an HPLC-PDA system (e2695 HPLC system and 2998 PDA detector, Waters, MA, USA), as follows: A C18 column (Symmetry, 4.6 × 150 mm, 5 μm) was used for the separation of the analytes, with column temperature adjusted at 25 °C, and a flow rate of 0.7 mL/min. The separation was achieved with isocratic elution, with a mobile phase consisting of 50% aqueous solvent (0.1% formic acid in H_2_O) (A) and 50% acetonitrile (B). The injection volume was 10 μL, and the total analysis time was 23 min. Calibration standards were prepared in the mobile phase in the range of 1 to 100 ppm, and the carvacrol response showed excellent linearity, with a coefficient of determination R^2^ > 0.99. The limit of quantification (LOQ) for carvacrol was estimated to be 0.13 mg/L. UV-Vis spectra were recorded from 210 to 400 nm, while the detection of carvacrol was performed at 280 nm. The retention time of carvacrol was 11.79 min (Figure 1).

### 3.5. Determination of Total Phenolic Content (TPC)

The determination of TPC was carried out by applying the Folin–Ciocalteu method, based on Dambolena et al. (2010), with some modifications [62]. A volume of 500 μL extract (or gallic acid standard, or water/ethanol mixture for blanks) was mixed with 500 μL of Folin–Ciocalteu reagent, and the mixture was shaken. Then 1 mL of Na_2_CO_3_ 1M solution was added, and the total volume was adjusted to 10 mL with deionized water. The mixture was then incubated for 60 min, and TPC was determined at 765 nm against a blank solution (containing 500 μL of solvent instead of extract) using a UV-Vis spectrophotometer (V-730, JASCO, Tokyo, Japan). TPC was expressed as Gallic Acid Equivalents, GAEs, per g of plant dry mass (mg GAE/g dm).

### 3.6. Determination of Total Flavonoid Content (TFC)

For the estimation of the TFC of the extracts, the aluminum chloride colorimetric method was applied [63]. Briefly, 0.1 mL of extract (or quercetin standard, or water/ethanol mixture for blanks) was mixed with 0.3 mL of 5% NaNO_2_ solution, and the mixture was allowed to stand for 5 min. Consequently, 0.3 mL of 10% AlCl_3_ was added, and after 6 min, another 2 mL of 1 M NaOH. The final volume was adjusted to 10 mL with ultrapure water. TFC was determined by the measurement of the absorbance of the final samples at 510 nm, using a UV-Vis spectrophotometer (V-730, JASCO, Tokyo, Japan). The content of total flavonoids was expressed in mg of quercetin equivalents per g of plant dry mass (mg QE/g dm).

### 3.7. Response Surface Analysis and Statistics

There is a significant lack of knowledge regarding the chemical composition of *Origanum dubium* alcoholic extracts, as relevant data are scarce in the literature. *Origanum vulgare* is a much better studied species; however, significant differences in the concentrations and types of secondary metabolites are expected in their extracts [64,65,66,67]. Response Surface Methodology (RSM) was selected in this work in order to determine the optimal extraction conditions for carvacrol, total phenolic, and flavonoid compounds of Origanum dubium leaves. RSM is a reliable statistical technique that is used in process optimization, as it allows the simultaneous evaluation of the combined effects of multiple independent variables on one or more responses and the determination of optimal conditions [55,61]. 

Evaluation of the experimental results and fitting of the full quadratic approximation of the BBD response model was carried out by analysis of variance (ANOVA), and the significance of regression coefficients was determined based on the obtained *p*-values. For each evaluated independent factor, a Prob > F-value of less than 0.05 indicated the significance of the response of the measured parameters (carvacrol content, TPC, TFC). Responses with *p*-values lower than 0.05 at the 95% confidence level were accepted as statistically significant. The lack of fit estimated by ANOVA was found to be 0.50, thus not significant (>0.05), which indicates that the model adequately fits the experimental data, and the variation not explained by the model is comparable to the experimental error. The estimation of the lack of fit is crucial when applying response surface analysis, as *p*-values below 0.05 for the lack of fit indicate that the use of this methodology would produce misleading results [68]. All statistics were carried out using the Origin Pro 2024 (v. 10.1.0.178) software.

## 4. Conclusions

In this study, a probe-type, ultrasonic-assisted green extraction method using ethanol–water mixtures as solvents was successfully optimized in terms of the extraction time, extraction temperature, ethanol–water ratio, and probe power amplitude, to provide the maximum yields for carvacrol, total phenolics, and total flavonoids from *Origanum dubium* plant leaves. A BBD was applied to design the set of experiments, and response surface analysis was used for the statistical evaluation of the significance of the impact of each independent extraction factor on the response parameters. The analysis determined the optimal extraction conditions, which provided a balanced maximization of carvacrol, TPC, and TFC.

The selected extraction technique, along with the optimal conditions, resulted in significant phenolic compound yields, reflecting the potential of this method to be applied as an efficient and sustainable choice for the extraction of bioactive compounds from *Origanum dubium* and other aromatic plants. The method combines green solvents and an extraction technique with relatively low extraction times and energy consumption, aligned with the principles of green chemistry, while providing *O. dubium* extracts enriched in phenolics. These extracts could be further explored and utilized in nutraceutical, pharmaceutical, or other applications.

## Figures and Tables

**Table 1:** Results of the analysis of variance (ANOVA) for the extraction of carvacrol, TPC and TFC.

Coefficient	Carvacrol		TPC		TFC	
	F-Value	*p*-Value	F-Value	*p*-Value	F-Value	*p*-Value
X1 time	50.63	0.000	0.109	0.745	2.40	0.139
X2 temperature	4.41	0.048	24.05	0.000	19.61	0.000
X3 ethanol ratio	77.41	0.000	2.03	0.171	18.51	0.000
X4 power	0.02	0.884	0.78	0.388	0.35	0.563
X1·X1	33.77	0.000	0.57	0.459	0.19	0.667
X2·X2	0.44	0.501	0.00	0.969	0.49	0.493
X3·X3	27.47	0.000	16.39	0.000	15.04	0.001
X4·X4	1.00	0.330	0.00	0.992	0.00	0.991
R^2^	0.913		0.735		0.765	
Adj R^2^	0.874		0.617		0.660	
Lack of fit (*p*-value)	1.378	0.500	10.90	0.089	4.182	0.210

**Table 2:** Box–Behnken Design showing actual and coded values of the four independent parameters and experimental results for total phenolic, total flavonoid, and carvacrol content of extracts.

	Independent Factors				Dependent Factors		
Run	Time (min)	Temp. (°C)	Ethanol (% *v*/*v*)	Power (%)	TPC (mg GAE/g dm)	TFC (mg QE/g dm)	Carvacrol (mg/g dm)
1	30 (+1)	50 (0)	60 (0)	50 (−1)	61.41	26.43	16.13
2	15 (0)	50 (0)	40 (−1)	100 (+1)	50.87	21.15	10.04
3	5 (−1)	70 (+1)	60 (0)	75 (0)	69.28	24.43	11.02
4	15 (0)	70 (+1)	60 (0)	100 (+1)	62.14	25.73	15.89
5	15 (0)	30 (−1)	40 (−1)	75 (0)	44.79	16.07	10.35
6	15 (0)	50 (0)	60 (0)	75 (0)	53.50	20.38	13.80
7	15 (0)	70 (+1)	40 (−1)	75 (0)	50.84	21.38	9.34
8	15 (0)	30 (−1)	60 (0)	100 (+1)	51.76	18.99	14.21
9	5 (−1)	50 (0)	80 (+1)	75 (0)	45.75	14.82	10.91
10	15 (0)	50 (0)	60 (0)	75 (0)	53.66	20.62	15.20
11	15 (0)	50 (0)	80 (+1)	100 (+1)	49.02	16.53	15.16
12	15 (0)	50 (0)	60 (0)	75 (0)	55.56	22.25	13.99
13	5 (−1)	50 (0)	40 (−1)	75 (0)	52.30	20.51	7.68
14	5 (−1)	30 (−1)	60 (0)	75 (0)	47.04	16.09	9.78
15	15 (0)	70 (+1)	60 (0)	50 (−1)	54.70	20.46	14.31
16	30 (+1)	50 (0)	60 (0)	100 (+1)	54.47	19.62	14.50
17	30(+1)	50 (0)	40 (−1)	75 (0)	49.53	20.13	8.50
18	30 (+1)	50 (0)	80 (+1)	75 (0)	47.94	14.87	14.75
19	15 (0)	30 (−1)	80 (+1)	75 (0)	43.83	13.70	13.71
20	15 (0)	70 (+1)	80 (+1)	75 (0)	49.99	16.15	15.06
21	5 (−1)	50 (0)	60 (0)	100 (+1)	52.40	18.27	10.47
22	30 (+1)	70 (+1)	60 (0)	75 (0)	58.43	22.91	15.10
23	30 (+1)	30 (−1)	60 (0)	75 (0)	50.53	18.78	12.89
24	15 (0)	50 (0)	80 (+1)	50 (−1)	44.83	13.99	14.34
25	5 (−1)	50 (0)	60 (0)	50 (−1)	51.43	17.97	10.39
26	15 (0)	30 (−1)	60 (0)	50 (−1)	46.67	16.94	13.37
27	15 (0)	50 (0)	40 (−1)	50 (−1)	50.66	20.45	11.27

**Table 3:** Optimal extraction conditions, depending on the targeted, optimized parameter, and comparison of the predicted and the experimental values using combined optimized conditions.

Optimized Parameter	Time (min)	Temperature (°C)	Ethanol (% *v*/*v*)	Power (%)
Carvacrol	17.5	70	71	100
TPC	30	70	58	100
TFC	30	70	53	100
Carvacrol, TPC, TFC	26	70	60	100
	**Predicted value**	**Experimental value**	**Relative** **error (%)**	
Carvacrol	15.62	14.93	4.40	
TPC	60.92	60.03	1.46	
TFC	24.04	23.78	1.08	

## Data Availability

The original contributions presented in this study are included in the article. Further inquiries can be directed to the corresponding author(s).
